# Gender differences and similarities in medical students’ experiences of mistreatment by various groups of perpetrators

**DOI:** 10.1186/s12909-017-0974-4

**Published:** 2017-08-14

**Authors:** Heidi Siller, Gloria Tauber, Nikola Komlenac, Margarethe Hochleitner

**Affiliations:** Medical University of Innsbruck, Gender Medicine Unit, Innrain 66, 6020 Innsbruck, Austria

**Keywords:** Mistreatment of students, Medical students, Psychological mistreatment, Medical university

## Abstract

**Background:**

Mistreatment of medical students during medical education is a widespread concern. Studies have shown that medical students report the most mistreatment compared to students of other study programs and that the prevalence of mistreatment peaks during clinical training. For this reason, a study was conducted to assess prevalence of mistreatment among medical students committed by various groups of people. The focus was to identify whether gender was associated with the experience of mistreatment. Additionally, students’ perception of university climate for reporting sexual harassment was assessed.

**Method:**

In the study 88 medical students (45 women, 43 men) participated. A modified version of the Questionnaire on Student Abuse was used to assess students’ experience of various types of mistreatment and associated distress during medical education. To explore factors that could be associated with this experience the organizational climate for reporting sexual harassment was assessed with the Psychological Climate for Sexual Harassment.

**Result:**

The most often cited perpetrators of mistreatment were strangers (79.5%), friends (75.0%) and university staff (68.2%). Strangers mostly committed psychological mistreatment and sexual harassment, whereas friends additionally engaged in physical mistreatment of medical students. The most common form of mistreatment conducted by university staff was humiliation of students. These kinds of psychological mistreatment were reported to be distressing (43%). Gender differences were found in the prevalence of mistreatment. Women experienced more sexual harassment and humiliation than did men. On the other hand, men experienced more physical mistreatment than did women. Women reported experiencing more distress from mistreatment experiences than did men and also more often reported being mistreated by university staff than did men. Women perceived a greater risk in reporting sexual harassment to the organization than did men.

**Conclusion:**

Mistreatment of female and male students should be focused on using a gender perspective because types of mistreatment can differ by gender. Additionally, interventions should include the societal level as there was a high prevalence of mistreatment perpetrated by strangers. Also the issue of trust in the university needs to be addressed and the organization is called on to visibly demonstrate that it represents and protects its students as well as its staff.

## Background

Student mistreatment in medicine is a widespread concern and has been increasingly studied since publication of the article by Silver and Glicken [1] on student mistreatment. Since then the question has been debated whether it is an innate characteristic of medicine to mistreat and humiliate its students. This is also underlined by a Finnish study that showed that medical students were exposed to the most mistreatment during their university training as compared to students in other study programs [2]. Even though this study shows that student mistreatment does not happen exclusively in medicine, it demonstrates that medical students are the most affected. Consequently, the question was posed whether medical students “misinterpret” suboptimal learning environment as mistreatment [3]. However, it was also found that medical students are not overly sensitive to mistreatment and that the reporting of mistreatment experiences is not the result of student misinterpretation [4].

In this context the type of treatment medical students perceive as mistreatment appears to be of even more interest. In a systematic review and meta-analysis more than half of the medical students surveyed had experienced mistreatment in the form of verbal, sexual or physical harassment, namely 69%, 33% and 9%, respectively [5]. Medical students reported in particular verbal mistreatment [5–9] during their medical education, such as humiliation [2], or negative remarks and being shouted at [2, 7]. Other forms of mistreatment included, even though to a lesser extent, sexual [2, 7, 10] and physical harassment [7, 9] as well as abuse of power [9]. Mistreatment was especially exerted by residents and clinical staff [5, 6, 9, 11], professors [7, 10], but also by fellow students [10]. Negative experiences seemed to peak when entering clinical internship and during residency [6, 8], but were also prevalent in undergraduate education [10, 12].

Mistreatment of medical students is discussed as relating to forming and creating students’ professional identity [13], but also as stemming from the androcentric hierarchical structure of medicine and thereby affecting women and men differently [14].

The investigation of gender differences has not yet been performed systematically and intensively [14, 15]. Thus, so far it can be noted that some studies have found no gender differences in mistreatment of medical students [9, 16], whereas others have [2, 10]. For example, female medical students are affected particularly in terms of experiencing sexual harassment [10]. In this context it was found that female medical students appear to have learnt how to deal and cope with inappropriate behavior on the part of male patients. However, they felt unprepared when encountering inappropriate behavior on the part of male supervisors [15].

Despite the limited studies on gender differences in student mistreatment, student mistreatment should also be discussed with regard to the androcentric culture of medicine and the numerical feminization of this formerly androcentric field [17]. The culture in an organization, such as hospitals, or medicine for that matter, can be defined by shared values and beliefs, mostly unconsciously engrained and historically grown. Thus, these beliefs and assumptions have developed over time [18, 19]. The historically grown embedding and the unconscious nature of these values and beliefs mean changes in organizational and medical culture are slow. The medical culture is connected to power, hierarchy as well as disrespectful, deprecating and competitive behavior in medicine [20]. The competitive atmosphere in medical training is prevalent not only among students and graduates, but also with regard to collaborating with other professions, e.g. nurses, midwives [21]. Competition in this sense relates to other health professionals downgrading medical students, thereby reinforcing power structures and hierarchical levels. It was also found that both men and women were reported as perpetrators in this context [12]. In this sense, mistreatment can be discussed in relation to non-human perpetrators, such as organizations, institutions and societal structure [22, 23]. In medicine such non-human perpetrators refer to the medical culture, thus power structures between medical and other health care professions as well as hierarchical structures within the medical discipline. In this sense non-human perpetration refers to reifying and reproducing structures that foster mistreatment.

Mistreatment has considerable effects on health and well-being, e.g. feeling stressed [8], psychosomatic consequences [10] and burnout [11]. However, formal reports of mistreatment of medical students remain scarce [9]. Trust in college support systems and feeling connected to the campus community are associated with a greater willingness to report threats [24]. This underlines the importance of feeling safe and protected in terms of trusting an organization to take action against mistreatment [25]. This also highlights the university’s role in sanctioning student mistreatment and creating a safe environment for students as well as for staff. To start doing so, medical educators were asked to improve students’ educational experiences [8, 26, 27]. Furthermore, a general plea was made to change the culture in medicine [5, 28].

It is necessary to understand the mechanisms of mistreatment and demonstration of power that medical students face in order to succeed in implementing sustainable and successful intervention strategies to fight mistreatment in medical education.

In this article a survey of medical students at one medical university in Austria was conducted to assess medical students’ experiences with mistreatment in its psychological, sexual and physical form. In particular this study sought to investigate 1) the extent of mistreatment exerted by various groups of potential perpetrators (university staff, fellow students, friends, (ex-)partners or patients and patients’ relatives) and thus how mistreatment is rooted in medical education or outside the university. 2) Gender differences in experiencing mistreatment were analyzed as there is only limited evidence of these in scholarly literature. 3) The medical university’s social climate for reporting sexual harassment was assessed to obtain a picture of the students’ sense of safety and their trust in the organization to prosecute mistreatment seriously and effectively. This questionnaire was chosen as a university policy on sexual harassment focusing explicitly on students was initiated in 2014 [29]. Even though other forms of mistreatment, such as physical and psychological mistreatment, are also not tolerated, these have not been made the subject of special policies, but are included in the university’s official bulletins. However, these policies do not explicitly focus on students, but pertain to all members of the university. Also analyzed was whether participation in self-defense training was associated with medical students’ perception of the university’s social climate for reporting and dealing with mistreatment. This study was conceptualized to acquire information on the severity of these issues as only little has been written about European medical students experiencing mistreatment.

## Methods

### Procedure

Medical students were recruited at compulsory lectures on Gender Medicine at a medical university in Austria. Of 109 students attending these lectures 88 (80.7%) participated in the study. The completed questionnaires were collected after the lectures. Participants were recruited at the end of the fifth year of their study program to ensure that they had already obtained some clinical experience during internships and come to know the medical culture at this medical university. The study was conducted during three months (April – June) of the summer term 2015.

### Instruments

#### Socio-demographic questions

Participants were asked their age, gender and relationship status.

#### Discrimination, harassment and mistreatment in medical students

The *Modified Version of the Questionnaire on Student Abuse* [2] was used to assess students’ experiences of mistreatment during medical education. The questionnaire consists of 38 items on physical, psychological mistreatment and sexual harassment and discrimination. Items were assessed on a four-point Likert scale (ranging from 1 = “*never*” to 4 = “*often*”) including open questions for specifying experienced mistreating acts. For each kind of mistreatment the students were asked who the perpetrator was. The list of possible perpetrators included (ex-)partners, patients and patients’ relatives, friends and strangers in addition to perpetrators who were fellow students or university staff. Additionally, students were asked how bothered or distressed they felt by each experience (answers ranged from 0 = “*didn’t experience this kind of mistreatment*”, 1 = “*was not distressed*”, 2 = “*somewhat distressed*” to 3 = “*was very distressed*”). Three questions on sleep deficits were excluded as they did not fit the purpose of the study.

#### Perceived climate for reporting sexual mistreatment

The *Psychological Climate for Sexual Harassment (PCSH) Questionnaire* [25, 30] refers to the organization’s intolerance of harassment. It includes two scales. The first scale assessed the risk perceived by students for reporting sexual mistreatment. The second scale asks to what extent students feel the organization takes reports about harassment and mistreatment seriously. It further assesses the perceived seriousness/actions of the organization in prosecuting harassment. The questionnaire is composed of nine items. Answers were assessed on a five-point Likert scale (1 = “*strongly disagree*” to 5 = “*strongly agree*”). The organization’s intolerance of harassment scale had a reliability of Cronbach’s α = .59 (three items), whereas the perceived seriousness of the organization in prosecuting harassment scale had a Cronbach’s α of .70 (six items). The questionnaire was used to provide an impression of the perceived organizational climate in supporting and fighting harassment when reporting incidents.

Participants were also asked if they had learned self-defense in the past. Additionally, participants were asked about the reasons for taking a self-defense course and whether they felt safer after taking such a course.

### Statistical analysis

Because of the small sample size and the non-normal distribution of data non-parametric tests were used for the analyses. Besides providing percentages for demographic data and experiences with mistreatment, group comparisons were made using the Mann-Whitney *U* test and Chi-square analysis. In this study the level of significance was set at α = .05, which is in agreement with the majority of studies in social and human sciences that use this cut-off point for significance [31–34]. In this way, any false hypotheses that state that no group differences exist were rejected with 95% confidence. Thus, all *p* values ≤ .05 were considered to be statistically significant.

Items asking about various forms of mistreatment experienced (shouting/yelling; humiliation; threats; hitting/kicking/shoving; sexual harassment; discrimination based on age or ethnicity; negative remarks) and perpetrators (fellow students; staff; friends; patients and their relatives; (ex-)partners; strangers) were dichotomized into never experienced (answer 1 = “*never*”) and experienced (answers 2 = “*seldom*”, 3 = “*sometimes*” and 4 = “*often*” were summarized into one category).

For each perpetrator group (fellow students; staff; friends; patients and their relatives; (ex-)partners; strangers) the number of students who were affected at least *seldom* by at least one of the various forms of mistreatment was counted. After determining this number of students, the percentage was calculated for each perpetrator group. In this way, the most often named perpetrators, who committed at least one act of student mistreatment, could be determined.

## Results

### Participants

Eighty-eight medical students (45 women, 43 men) with a mean age of *M* = 24.7 (*SD =* 2.0; range 21–32) years participated in the study. Of the participants, 62.5% reported being in a partnership.

### Prevalence of mistreatment

The three groups perpetrating the most mistreatment of medical students were strangers (79.5%), friends (75.0%) and university staff (68.2%). Of the respondents 59.1% reported that mistreatment was committed by (ex-)partners and 58.0% stated that mistreatment was perpetrated by fellow students, whereas 47.7% reported mistreatment by patients.

An overview of the types of mistreatment committed by the three groups reported most often as perpetrators can be found in Table 1. In the following analyses only mistreatment reported by more than 25% of the students will be discussed in the remainder of the Results (see Table 1). It was observed that strangers were involved in a large number of mistreating behaviors, such as shouting or yelling at a student (61.4%), threatening a student with harm (37.5%), humiliating a medical student (31.8%), making negative or devaluing remarks about a student’s future profession (30.7%), and discriminating her/him because of ethnicity, religion or age (28.4%). In addition to psychological mistreatment, strangers were also named in the context of sexual harassment (35.2%).

**Table 1:** Prevalence of mistreatment acts committed by the three most reported perpetrator groups

	Perpetrators: Strangers			Perpetrators: Friends			Perpetrators: University staff		
	All %	W %	M %	All %	W %	M %	All %	W %	M %
Mistreatment act									
Shouting or yelling	61.4	64.4	58.1	60.2	55.6	65.1	29.5	35.6	23.3
Threatening with harm	37.5	31.1	44.2	13.6	11.1	16.3	3.4	2.2	4.7
Humiliation	31.8	35.6	27.9	40.9	46.7	34.9	40.9	51.1	30.2
Negative or devaluing remarks about future profession	30.7	26.7	34.9	28.4	28.9	27.9	23.9	26.7	20.9
Discrimination on the basis of (ethnicity, religion or age)	28.4	33.3	23.3	21.6	15.6	27.9	22.7	22.2	23.3
Sexual discrimination and harassment	35.2	55.6	14.0	19.3	20	18.6	17.0	26.7	7.0
Hitting, shoving, kicking	18.2	13.3	23.3	27.3	15.6	39.5	1.1	0.0	2.3

*N* = 88 (45 female, 43 male) medical students. *W* Women, *M* Men

Medical students also reported friends as being abusive in various ways and being perpetrators not only of psychological forms of abuse (shouting/yelling 60.2%, humiliation 40.9%, negative remarks about the student’s future profession 28.4%), but also of physical mistreatment (hitting, shoving or kicking 27.3%).

University staff members exerted power over medical students and most often committed mistreatment by humiliating (40.9%) students.

### Distress from mistreatment

Irrespective of the perpetrators, the most stress was experienced by being shouted at or yelled at (somewhat or very stressful for 43.2% and 10.2% of students, respectively) and by being humiliated (somewhat for 34.1%, very stressful for 15.9%). Sexual harassment was experienced as stressful by almost 30% of students (somewhat stressful 25.0%, very stressful 4.5%). Other forms of mistreatment were experienced as somewhat or very stressful by less than 25% of the medical students (see Table 2)

**Table 2:** Perceived distress caused by acts of mistreatment

	Mistreatment act perceived as distressing			
	All %	W %	M %	χ^2^(1); *p*
Mistreatment act				
Shouting or yelling	53.4	60.0	46.5	2.37; .124
Threatening with harm	19.3	24.4	14.0	1.05; .305
Humiliation	50.0	68.9	30.2	11.44; .001
Negative or devaluing remarks about future profession	18.2	20.0	16.3	0.08; .777
Discrimination on the basis of (ethnicity, religion or age)	22.7	28.9	16.3	1.52; .218
Sexual discrimination and harassment	29.5	40.0	18.6	4.00; .046
Hitting, shoving, kicking	17.0	20.0	14.0	0.43; .514

*N* = 88 (45 female, 43 male) medical students. *W* Women, *M* Men

### Gender differences

Women were more likely to be exposed to harassment and sexual mistreatment (68.9%) than were men (32.6%) (χ^*2*^(1) = 11.6, *p* = .001). Men were significantly more often subjected to hitting, kicking or shoving (48.8%) than were women (24.4%) (χ^*2*^(1) = 5.7, *p* = .017). More women (77.8%) than men (53.5%) reported having experienced humiliation (χ^*2*^(1) = 5.8, *p* = .016). No other forms of mistreatment evidenced significant gender differences (all χ^*2*^(1) < 1.6, *ps* > .207).

More women (68.9%) than men (30.2%) perceived humiliating experiences as distressing (χ^*2*^(1) = 11.44, *p* = .001). Further, more women (40.0%) perceived harassment and sexual mistreatment as distressing than did men (18.6%) (χ^*2*^(1) = 4.0, *p* = .046). No other significant gender differences in reported distress were found (Table 2).

Overall, reported groups of perpetrators did not differ between women and men (all χ^*2*^(1) < 1.1, *ps* > .290), except for one group. Namely, women (77.8%) reported more often being mistreated by university staff than did men (58.1%) (χ^*2*^(1) = 3.9, *p* = .048).

Women perceived a greater risk for reporting sexual harassment (*Mdn* = 3.3, interquartile range [IQR] = 2.8–3.7) than did male students (*Mdn* = 4.3, IQR = 3.6–4.7) (*U* = 446, *z* = − 4.28, *p* < .001). Men having experienced any kind of mistreatment by university staff (*Mdn* = 4.0, IQR = 3.3–4.3) perceived a greater risk for reporting sexual harassment than did men who did not undergo such experiences (*Mdn* = 4.3, IQR = 3.7–4.7) (*U* = 139, *z* = −2.0, *p* = .047). Women who experienced mistreatment by fellow students were less likely to believe that the organization would take action against mistreatment or view it as a serious issue (*Mdn* = 3.2, IQR = 2.6–3.6) than were women who did not undergo such experiences (*Mdn* = 3.6, IQR = 3.2–4.0) (*U* = 143, *z* = −2.24, *p* = .025). With regard to other groups of perpetrators, no significant gender differences were found concerning risk entailed in reporting harassment or belief that action would be taken consequent to reporting harassment.

### Social climate for reporting harassment at the university

Overall, students reported perceiving risks when considering reporting sexual harassment (*Mdn* = 3.7, IQR = 3.0–4.3). However, students overall thought that such a report would be taken seriously (*Mdn* = 3.4, IQR = 3.0–3.8).

Of the participants 33% had attended a minimum of one and a maximum of ten self-defense training sessions. As many women (40.0%) as men (25.6%) had taken self-defense training (χ^*2*^(1) = 2.07, *p* = .150). The majority of students (86.2%) who attended a self-defense course did not base their decision to take the course on any prior incident experienced either by themselves or by someone else, or on media news reports. Of those who attended self-defense training 58.6% reported feeling safer and better prepared to fight off aggressors after learning self-defense. There were no significant gender differences regarding these aspects (χ_reason_^2^(2) = .79, *p* > .999; χ_safety_^2^(1) = .18, *p* = .717).

Those students who attended self-defense training more often reported experiencing sexual harassment (44.4%) than did those who did not learn self-defense (20.9%) (χ^*2*^(1) = 5.5, *p* = .024). No significant differences regarding other forms of mistreatment and no differences between attendees and non-attendees were found (all χ^*2*^(1) < 0.9, *ps* > .475).

Overall, attending a self-defense course was associated neither with perceiving a risk in reporting sexual harassment (*U* = 715, *z* = −1.01, *p* = .311) nor with the respondent’s perception of how serious the organization takes reports of sexual harassment (*U* = 737, *z* = −0.81, *p* = .416). When analyzing these relationships separately by gender, attending a self-defense course was associated with the perception by women of how serious the organization takes reports of sexual harassment (*U* = 159, *z* = −1.96, *p* = .050), but not by men (*U* = 111, *z* = −1.46, *p* = .154). Women who took a self-defense course perceived the organization to take reports of sexual harassment less seriously (*Mdn* = 3.0, IQR = 2.4–3.7) than did women who did not take such a course (*Mdn* = 3.6, IQR = 3.2–3.8). The risk perceived in reporting sexual harassment was not associated with taking a self-defense course for women (*U* = 191, *z* = −1.24, *p* = .216) or for men (*U* = 113, *z* = −1.42, *p* = .163).

## Discussion

This study focused on mistreatment in medical students. The scientific literature reports that medical students experience various forms of mistreatment, especially from clinical [5, 9] and university staff [7, 8, 10]. In particular shouting, yelling and humiliation appear to be prevalent [2, 7]. The current study replicated these results. Students also reported being distressed by having experienced such forms of mistreatment. Especially female medical students appear to suffer sexual harassment. This was also shown in a German study [8], a Dutch study [35] and a study conducted in California [9]. In contrast to the studies reported in the literature, the current study shows that sexual harassment was hardly perpetrated by staff. It was committed mostly by strangers. This finding is also in line with another study that differentiated between stranger and non-stranger sexual harassment [36]. It was found that sexual harassment of women was more commonly committed by strangers than by non-strangers [36].

Female medical students reported more mistreatment by university staff than did male students. Even though this finding is not surprising, it illustrates that such discrimination and mistreatment pervades the career of women in medicine, already starting in medical education [37]. Despite the fact that equal numbers of women and men enter medical studies, women are still subjected to more mistreatment by staff. Such abuse underlines the need to change the medical culture, and also its apparent gendered basis should be challenged. Additionally, the greater incidence of humiliation of women compared to men as found in this study was also found in other studies [2] and is worrisome. These findings emphasize the organization’s need to react accordingly and implement improved strategies to ensure not only the physical but also the emotional safety of students and staff. The strategies implemented so far are evidently nowhere near being sustainable and efficient enough. The organization’s need to react to mistreatment can also be perceived in students’ distrust of the organization and the risk they perceive in reporting harassment.

Men subjected to mistreatment reported that they perceived greater risk in the reporting of sexual harassment by university staff than did men who did not experience mistreatment. Moreover, women in general perceived a greater risk in reporting sexual harassment than did men. This might be connected to power structures and fear of being hindered in one’s career by members of the organization after reporting abuse. Additionally, it can be concluded that there prevails a lack of trust in the university’s ability to protect students from negative effects on their future careers as a result of reporting mistreatment. Furthermore, the question begs to be asked whom the university represents and whom the university protects. As found in the current study, female students who had been mistreated by fellow students did not believe the university would perceive this mistreatment as a serious issue or one worth prosecuting. Thus, the university might give the impression that it hardly protects students. Also, self-defense courses offered by the university did not appear to increase trust in the organization to prosecute mistreatment. This impression seemed prevalent, especially among women mistreated by fellow students. Consequently, one conclusion might be that the university does not represent and protect students as well as staff of all genders.

In addition, humiliation by university staff might be influenced by the hierarchical structures in medicine. It could be argued that such forms of mistreatment result from a misconception that medical students need to be prepared for the rough hierarchical structures of the medical profession. Accordingly, the medical culture provides the breeding ground where students are “taught” how to treat persons of lower rank and status. This demonstration of power (e.g. staff demonstrating power over students) in medicine might result in a transgenerational legacy [27], in which these practices are passed from university staff to medical students and might also reinforce mistreating behavior in future generations of medical doctors [27, 38]. It is suggested that incidences and prevalence of mistreatment not only be studied, but also be brought in association with power relationships and hierarchical structures.

Overall, mistreatment was not only committed by university staff, but also by friends and strangers. Hart and Miethe [39] pointed out that mistreatment is more commonly found off campus than on campus. The context of experiencing mistreatment off campus could also be an influential variable in the current study. The reporting of strangers involved in mistreating behavior towards medical students could be attributed to off-campus experiences. Notably, in the current study a differentiation between on-campus and off-campus (or more appropriately inside and outside the university) experiences was not included in this study. However, it has to be noted that campuses are not prevalent in Austria. The university buildings are located in the city and are not surrounded by a campus area. Thus, this social environment might affect students’ exposure to mistreatment [40] and partially explain why this study showed perpetrators to be strangers or friends.

The current study has several limitations. It was conceptualized as a first attempt to gain information on the severity of mistreatment of medical students at this medical university. Thus, comparison with a control group of students from other disciplines and a non-student population was not sought at this point. Additionally, the small sample size does not allow generalization for all medical students at this medical university. Another limitation includes the issue of self-selection by participants. Even though a response rate of 80% was achieved, no information was available on those students who did not participate in the study. Thus, the participating students might be especially aware of the issue of mistreatment perpetrated against students and might have experienced more mistreatment than did those who did not participate in the study. In this sense, participating students were more willing to share their experiences.

## Conclusions

The study offers important insights into mistreatment experienced by medical students. Mistreatment of medical students should be focused on using a gender perspective because types of mistreatment can differ by gender. These gender differences should also be viewed with regard to power relations and hierarchical structures, as these are considered to have an influence on mistreatment. Moreover, interventions to reduce and eliminate student mistreatment should not be restricted to the university as there was a high prevalence of mistreatment perpetrated by strangers. Intervention and campaigns should thus also include the societal level in which students operate, especially as this study shows that mistreatment is not only limited to mistreatment practiced behind the university walls, but that it is a reality for students in general. In order to do so and to enhance trust in the university’s fight against mistreatment, especially for all members of the university, it appears to be necessary to make university policies and actions against harassment and mistreatment in general more visible (e.g. publishing policies on the front page of the university website, displaying folders and posters on this matter at the university, announcing activities such as talks and workshops on this matter, training university staff in non-humiliating ways to teach students). Additionally, it appears to be vital to address the issue of trust in the university and the university’s apparent failure to protect students. Thus, the university has the duty to represent and protect all staff and all students.
